# Supplementary figures and images for: Dihydromyricetin inhibits injury caused by ischemic stroke through the lncRNA SNHG17/miR-452-3p/CXCR4 axis
Source: PeerJ. 2025 Jan 29;13:e18876. doi: 10.7717/peerj.18876 (PMC11786715; doi:10.7717/peerj.18876)

Fig. 6F

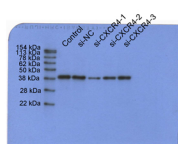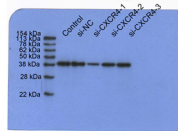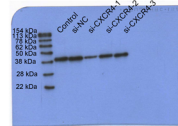

**CXCR4 41kDa**

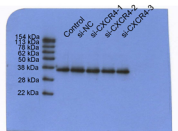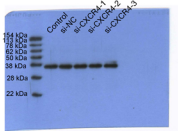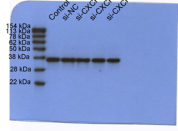

**GAPDH 37kDa**

Fig.8C

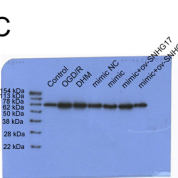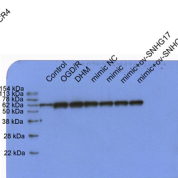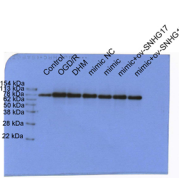

**p-NF-κB 65kDa**

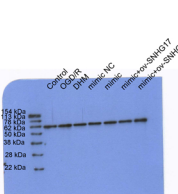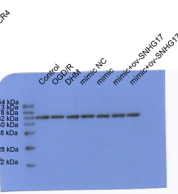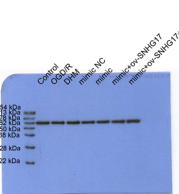

**NF-κB 65kDa**

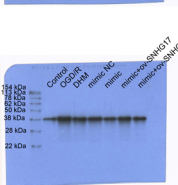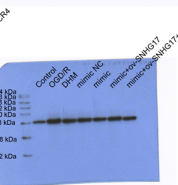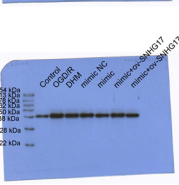

**CXCR4 41kDa**

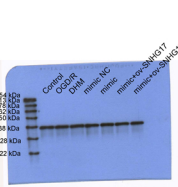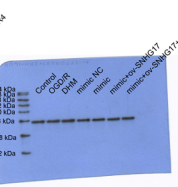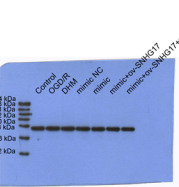

**GAPDH 37kDa**

Supplement: Supplemental Information 1 [file peerj-13-18876-s001.pdf]
