# Supplementary material for: Dihydromyricetin inhibits injury caused by ischemic stroke through the lncRNA SNHG17/miR-452-3p/CXCR4 axis
Source: PeerJ. 2025 Jan 29;13:e18876. doi: 10.7717/peerj.18876 (PMC11786715; doi:10.7717/peerj.18876)
Supplement: Supplemental Information 2 [file peerj-13-18876-s002.docx]

**MIQE Checklist**

| **ITEM TO CHECK** | **IMPORTANCE** | **CHECKLIST** |
| --- | --- | --- |
| **EXPRIMNETAL DESIGN** | | |
| Definition of experiment and control group | E | Page 4, lines 95–104 |
| Number within each group | E | Page 4, lines 102–104 |
| Assay carried out by core lab or investigator's lab? | D | Page 7, lines 187–188 |
| Acknowledgement of author's contributions | D | Page 12, lines 378–384 |
| Description | E |  |
| Volume /mass of sammples processed | D | N/A |
| Microdissection or macrodissection | E | N/A |
| Processing procedure | E |  |
| If frozen-how and how quickly? | E | N/A |
| If fixed-with what how quickly? | E | N/A |
| Sample storage conditions and durarion especially for ffpe sample | E | N/A |
| **NUCLEIC ACID EXTRACTION** | | |
| Procedure and/or details of any modifications | E |  |
| Name of kit and details of any modifications | E | Page 5, lines 141–149 |
| Source of additional reagents used | D | Page 5, lines 141–149 |
| Details of DNase or RNase treatment | E | Page 5, lines 141–149 |
| Contamination assessment (DNA or RNA) | E | Page 5, lines 141–149 |
| Nucleic acid quantification | E |  |
| Instrument and method | E | Page 5, lines 143–145 |
| Purity(A260/A280) | D | Page 5, lines 143–145 |
| Yield | D | N/A |
| RNA integrity method/instrument | E | Page 5, lines 143–145 |
| RIN/RQI or Cq of 3’and 5'transcripts | E | Page 5, lines 143–145 |
| Electrophoresis traces | D | N/A |
| Inhibition testing (Cq dilutions, spike or other) | E | N/A |
| **REVERSE TRANSCRIPTION** | | |
| Complete reaction conditions | E | Page 5, lines 146–148 |
| Amount of RNA and reaction volume | E | Page 5, lines 146–148 |
| Priming oligonucleotide and concentration | E | Table 1 |
| Reverse transcriptase and concentrarion | E | Page 5, lines 146–155 |
| Temperature and time | E | Page 5, lines 146–155 |
| Manufacture of reagents and catalpgue numbers | D | Page 5, lines 146–155 |
| Cqs with and without RT | D | Page 5, lines 146–155 |
| Storage conditions od cDNA | D | Page 5, lines 146–155 |
| **qPCR TARGET INFORMATION** | | |
| If multiplex efficiency and LOD of each assay | E | N/A |
| Sequence accession number | E | N/A |
| Location of amplicion | D | N/A |
| Amplicon length | E | N/A |
| *In silico* specificity screen | E | N/A |
| Pseudogenes,retropsendogenes or other homologs | D |  |
| Sequence alignmen | D |  |
| Secondart structure analysis of amplicon | D | N/A |
| Location of each primier by exon or intron | E | N/A |
| What splice variants are targeted | E | N/A |
| **qPCR OLIGONUCLEOTIDES** | | |
| Primer sequences | E | Table 2 |
| Rtprimer DB Identification number | D | N/A |
| Probe sequence | D | N/A |
| Location and identity of any modifications | E | N/A |
| Manofacture of oligonuclortides | D | Page 5, lines 146–149 |
| Purification method | D | N/A |
| **qPCR PROTOCOL** | | |
| Complete reaction conditions | E | Page 5, lines 140–143 |
| Reaction volume and amount of cDNA/DNA | E | Page 5, lines 146 |
| Primer,(probe),mg++,and dNTP concentrations | E | N/A |
| Polymerase identity and concentration | E | Page 5, lines 140–145 |
| Buffer/kit identity and manufacturer | E | Page 5, lines 146–149 |
| Exact chemical constitution of the buffer | D | N/A |
| Additives (SYBRGREENI,DMSO) | E | N/A |
| Manufacture of plates/tubes and catalog number | D | Page 5, lines 146 |
| Complete thermocycling parameters | E | Page 5, lines 147–152 |
| Reaction setup (manual/robotic) | D | N/A |
| Manufacture of qpcr instrument | E | Page 5, lines 148–153 |
| **qPCR VALIDATION** | | |
| Evidence of optimasation(from gradients) | D | N/A |
| Specificity(gel,sequence,melt,or digest) | E | N/A |
| For SYBR green GREEN I,cq of the NTC | E | Page 5, lines 146–150 |
| Standard curves with slope and y-intercept | E | Page 5, lines 146–150 |
| PCR effiency calculated from slope | E | N/A |
| Confidence interval for pcr efficiency or standard error | D | Page 5, lines 146–150 |
| r2 of standard curve | E | Page 5, lines 146–150 |
| Linear dynamic range | E | N/A |
| Cq variation at lower limit | E | N/A |
| Confidence intervals throughout range | D | N/A |
| Evidence for limit of detection | E | N/A |
| If multiplex efficiency and LOD of each assay | E | N/A |
| **DATA ANALYSIS** | | |
| qPCR analysis program (source,version) | E | N/A |
| Cq method determination | E | Page 5, lines 153 |
| Outlier identification and disposition | E | N/A |
| Result of NTCs | E | N/A |
| Justification of number and choice of reference genes | E | Page 5, lines 154 |
| Description of normalisation method | E | Page 5, lines 154-155 |
| Number and concordance of biological replicates | D | Page 5, lines 154 |
| Number and stage (RT or qpcr)of technical replicates | E | N/A |
| Repeatability (intra assay variation,) | E | N/A |
| Repeatability (intra assay variation,%CV) | D | N/A |
| Power analysis | D | N/A |
| Statistical methods for result significance | E | Page 7, lines 187-191 |
| Software(source,version) | E | Page 5, lines 149-151 |
| Cq or raw data submission using RDML | D | N/A |
